# Radiofrequency ablation for Barrett’s oesophagus related neoplasia with the 360 Express catheter: initial experience from the United Kingdom and Ireland—preliminary results

**DOI:** 10.1007/s00464-021-08325-0

**Published:** 2021-02-05

**Authors:** Cormac G. Magee, David Graham, Charles Gordon, Jason Dunn, Ian Penman, Robert Willert, Howard Smart, Jacobo Ortiz-Fernandez-Sordo, Krish Ragunath, Martin Everson, Durayd Alzoubaidi, Matthew Banks, Danielle Morris, Sarmed Sami, Allan J. Morris, Pradeep Bhandari, Ravi Narayanasamy, Massimiliano Di Pietro, Laurence B. Lovat, Rehan Haidry

**Affiliations:** 1 University College London Hospital, London, UK; 2 Centre for Obesity Research, University College London, London, UK; 3 Royal Bournemouth and Christchurch Hospitals, Bournemouth, UK; 4 Guy’s and St Thomas’ Hospital, London, UK; 5 Royal Infirmary of Edinburgh, Edinburgh, UK; 6 Manchester Royal Infirmary, Manchester, UK; 7 Royal Liverpool University Hospital, Liverpool, UK; 8 Nottingham Digestive Diseases Centre, NIHR Biomedical Research Centre, Nottingham University Hospitals NHS Trust, Nottingham, UK; 9 Division of Surgery and Interventional Sciences, University College London, London, UK; 10 Portsmouth University Hospital, Portsmouth, UK; 11 St James’ Hospital, Dublin, Ireland; 12 Glasgow Royal Infirmary, Glasgow, UK; 13 Cambridge University Hospitals, Cambridge, UK

**Keywords:** Barrett’s oesophagus, radio-frequency ablation, Strictures, Early oesophageal neoplasia, Express device

## Abstract

**Background:**

Radio-frequency ablation (RFA) for Barrett’s oesophagus (BE)-related neoplasia is currently used after endoscopic resection of visible neoplasia. The HALO 360 balloon has been used to ablate long segment BE. The Barrx™ 360 Express RFA self-sizing catheter (‘RFA Express’) may potentially allow quicker ablation times and improved treatment outcomes. The aim of this paper is to present real world data on the use of the 360 Express Device.

**Methods:**

Centres in the UK and Ireland submitted cases where the RFA Express was used. The primary outcome was regression of BE at 3 months. Secondary outcomes were the rate of symptomatic stricture formation and resolution of intestinal metaplasia (CR-IM) and dysplasia (CR-D) at End of Treatment (EoT).

**Results:**

11 centres submitted 123 consecutive patients. 112 had a follow up endoscopy. The median age was 67 years (IQR 62–75). 3 dosimetries were used. The mean reduction in Circumferential (C) length was 78% ± 36 and mean reduction in Maximal length (M) was 55% ± 36. 17 patients (15%) developed strictures requiring dilation. There was a higher rate of stricture formation when the 12 J energy was used (p < 0.05). 47 patients had EoT biopsies, 40 (85%) had CR-D and 34(76%) had CR-IM.

**Conclusions:**

The RFA 360 Express catheter shows reduction in length of baseline BE at 3 months after index treatment, and eradication of intestinal metaplasia and dysplasia at 12 months similar to other studies with earlier devices. It appears that the symptomatic stricture rate is slightly higher than previous series with the HALO 360 catheter.

This study was performed as part of the HALO registry and has been approved by the Research Ethics Committee - MREC Number 08/H0714/27 Local project reference 08/0104 Project ID 15,033 IRAS Number 54678 EudraCT 2009-015980-1. Registered on ISRCTN as below: ISRCTN93069556. https://doi.org/10.1186/ISRCTN93069556

Endoscopic eradication therapy (EET) for Barrett’s oesophagus related neoplasia is now well established [1] as the preferred strategy to surveillance or surgery in patients with mucosal neoplasia. There are multiple different field ablation techniques which can be used for the treatment of flat Barrett’s oesophagus related neoplasia after ER (Endoscopic Resection) of visible neoplasia. After Endoscopic Mucosal Resection (EMR) [2] or Endoscopic Submucosal Dissection (ESD) [3] field ablation of the residual BE with Photodynamic Therapy (PDT) [4], Argon Plasma Coagulation (APC) [5], Cryoablation [6] and Radio-Frequency Ablation (RFA) [7] have been used to reduce the risk of metachronous neoplasia arising.

International management guidelines recommend ER for the treatment of visible, dysplastic lesions followed by RFA for surrounding BE or for flat dysplasia [8–10].

In the UK and Ireland, the treatment protocol for BE related neoplasia constitutes initial removal of visible neoplastic lesions via endoscopic resection. The protocol for RFA following this is shown in Fig. 1, with endoscopies planned at 3 monthly intervals with further RFA treatment given when there is visible Barrett’s or Barrett’s seen on biopsies. End of treatment (EoT) biopsies are then taken at 12 months to assess for the complete resolution of intestinal metaplasia (CR-IM) and complete resolution of dysplasia (CR-D).

**Fig. 1:**
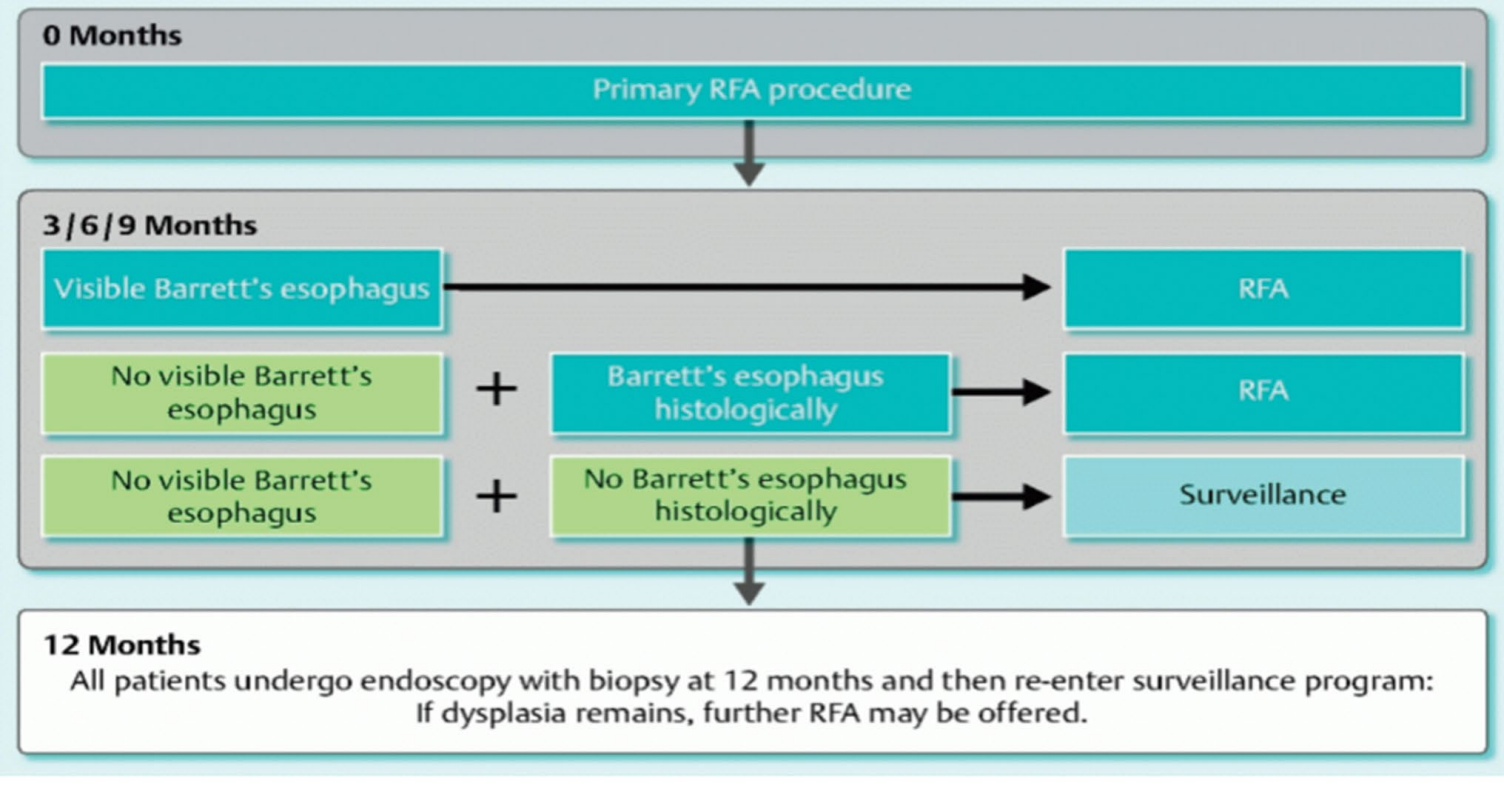
UK and Ireland treatment protocol for RFA in dysplastic Barrett’s oesophagus. (Used with permission of the HALO registry)

The Barrx™ system of RFA uses electrodes to deliver controlled radio-frequency pulses to the oesophageal mucosa at pre-set energy and power densities. This causes thermal injury and tissue destruction sparing the submucosa and reducing the risk of luminal narrowing and stricture formation due to disruption of the collagen matrix and submucosal layers [11]. A range of different catheters have been developed for RFA treatment of the oesophagus [12]. In patients with a longer circumferential segment of BE, a catheter mounted balloon with circumferential electrodes is used for the initial ablation to allow a larger surface area to be treated in a single session [13].

The previous RFA 360 Balloon catheter system consisted of sizing and treatment balloons. The oesophagus was initially sized with the sizing balloon and then reintubation was performed with an ablation catheter.

The standard ablation regimen used in the majority of studies and treatment protocols to date consists of initial ablation at 12 J/cm^2^ along the length of the BE under direct vision with the endoscope. The endoscope and the catheter are then removed, and a distal attachment cap is placed onto the endoscope and necrotic debris is removed/cleaned with this cap and subsequently water is flushed through the endoscope. The 360 catheter balloon is then placed over a guidewire before reintubation with the endoscope and a further ablation phase is performed at 12 J/cm^2^.

Two alternative treatment protocols have been used to simplify and streamline the circumferential RFA treatment of BE [14]. The first of these is the “simple with clean regimen” that involves attachment of the distal cap prior to insertion of the ablation catheter, thereby reducing the number of endoscopic intubations. The step of flushing water in the standard regimen is not performed. The second is the “simple no clean regimen” which involves immediate application of the second ablation following the first without any cleaning phase. A randomised study showed non-inferiority of this method [14] and this therefore became the preferred regimen due to reduced procedure time and reduced number of intubations. In both these regimens the dosimetry is 12 J/cm^2^.

The new HALO 360 Express catheter consists of a self-sizing balloon catheter which is 4 cm in length. As such, a longer segment of BE can potentially be ablated in fewer ablations by avoiding the need for pre-sizing of the oesophagus. The self-sizing catheter also reduces the number of intubations which may be more comfortable for the patient and reduce time. In addition, this should allow more uniform ablation as the oesophagus is sized at each ablation zone which should reduce the impact on mucosal contact of variations in OID (oesophageal internal diameter). This has been shown to reduce procedure time in another study [15]. The differences between the devices are shown in Table 1. The major differences between the two devices are that the HALO 360 Express Balloon has a longer length allowing more of the mucosa to be ablated, but also “self sizes” to the internal diameter of the oesophagus rather than the previous balloon which needed the internal diameter of the oesophagus to be measured with a different balloon first. The device is marketed as allowing a more rapid procedure but we wanted to investigate if there might be other outcome differences vs the previous balloon also. The instruction for use (IFU) from Medtronic states that the dosimetry and treatment protocol for the new device used in clinical practice should be 10 J/cm^2^ and that a cleaning phase a distal attachment cap followed by water irrigation should be performed when the device is used for the treatment of Barrett’s oesophagus [16]. Despite this many endoscopists, as seen in our data, used different regimens due to experience and published evidence with the earlier device as mentioned above. This could have an impact on outcomes and we discuss this later also.

**Table 1:** Differences between previous Barrx™ 360 catheter and Barrx™ 360 express catheter

Barrx™ 360 catheter	Barrx™ 360 express catheter
3 cm length	4 cm length
Sizing Balloon	No sizing balloon
2 intubations with sizing balloon and then treatment catheter	Single intubation with self-sizing catheter
Fixed balloon size which does not allow changes in balloon diameter through the Barrett’s segment	Allows for variable diameters in oesophagus

The aim of this study was to retrospectively evaluate the efficacy and safety (in terms of stricture formation) of the BARRx™ 360 Express RFA balloon catheter across specialist centres in the UK and Ireland after its initial limited launch in routine clinical practice, thereby presenting real world data on the use of this catheter. These were the only centres using the device at this time.

## Methods

The primary outcome was the surface regression of Barrett’s oesophagus at 3 months as calculated by % change in Circumferential (C) (the length from the GOJ to the highest point at which the Barrett’s mucosa is circumferential around the oesophagus) and Maximal (M) length (the length from the GOJ to the highest point at which the Barrett’s mucosa is in the oesophagus, e.g., as in a tongue of Barrett’s oesophagus). This was assessed by the reports of the follow up endoscopies performed by the endoscopists who had performed the initial procedure.

Secondary outcomes were resolution of intestinal metaplasia (CR-IM) and dysplasia (CR-D) at End of Treatment (EoT) and the rate of symptomatic stricture formation following RFA treatment.

The treatment dosimetry protocol was decided by the treating clinician.

Specialist centres in the UK and Ireland were invited to submit all consecutive cases meeting the below criteria.

## Inclusion criteria

1. Diagnosed with Barrett’s Oesophagus with intra-mucosal cancer, high grade dysplasia or low grade dysplasia.
2. Visible lesions removed by endoscopic resection (ER) prior to RFA.
3. Treated with the new BarrxTM 360 Express catheter as index RFA treatment
4. 3-month follow up endoscopy performed as minimum follow up

## Exclusion criteria

1. Previous oesophageal surgery
2. Previous radio-frequency ablation for Barrett’s oesophagus
3. Oesophageal strictures which would not allow passage of endoscope or balloon
4. Oesophageal varices

## Statistical analysis

Discrete variables are presented as medians with interquartile ranges (IQR) and continuous variables are presented as means with standard deviations (SD). Statistical analyses were performed with one way ANOVA, and Fisher’s Exact Test using GraphPad Prism for Mac v8.0.

## Ethical approval

This study was performed as part of the UK HALO registry and has been approved by the Research Ethics Committee – MREC Number 08/H0714/27 Local project reference 08/0104 Project ID 15,033 IRAS Number 54678 EudraCT 2009–015,980-1.

## Results

123 patients were submitted across 11 specialist centres in the UK and Ireland which were the first to use the 360 Express catheter in clinical practice after its limited clinical launch from November 2015 to November 2017.

The patient characteristics and the characteristics of the Baseline Barrett’s oesophagus are shown in Table 2.

**Table 2:** Patient characteristics and baseline Barrett’s oesophagus

Number of patients	123
Median age (years)	67 (IQR 62–75)
Male	102 (83%)
Female	21 (17%)
Low grade dysplasia	43 (35%)
High grade dysplasia	62 (51%)
Intra-mucosal carcinoma	18 (14%)
Mean BE circumferential (C) length cm	5 (IQR 2–8)
Mean BE maximal (M) length cm	8 (IQR 5–10)
Previous endoscopic resection	54 (44%)

11 patients were excluded as they had not reached 3-month endoscopic follow up at the time of this analysis.

**Fig. 2:**
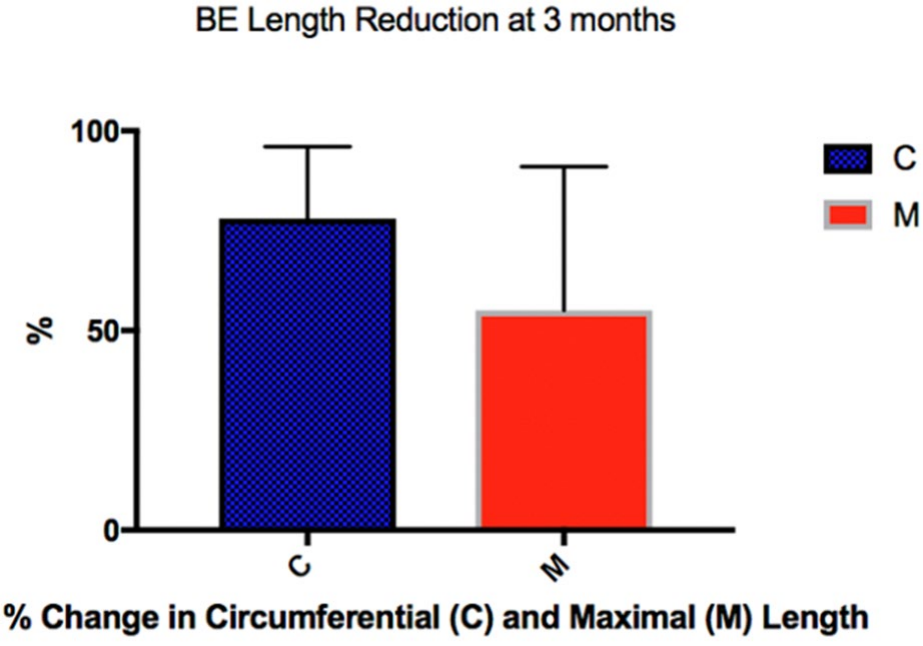
Mean percentage change in circumferential (C) and maximal (M) length of Barrett’s oesophagus at 3 months following treatment with RFA Express Catheter

At the first follow up endoscopy following RFA Express treatment (3 months post treatment) the mean % change in circumferential length (C) was 78 ± 36%. The mean % change in maximal length (M) was 55 ± 36%. (Fig. 2) There was no significant difference in the change in C and M when the three treatment protocols were compared. One way ANOVA p = NS.

**Fig. 3:**
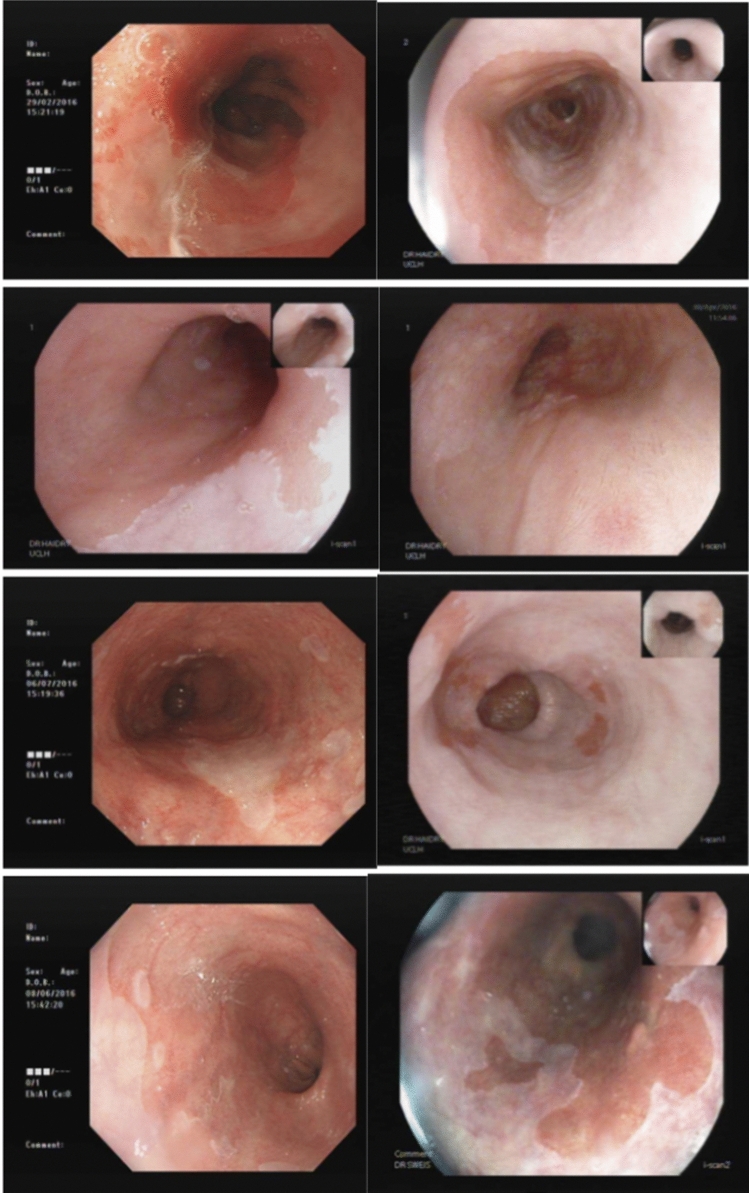
Endoscopic images of four patients before and after treatment with RFA Express and at first follow up endoscopy. Before treatment on left and after treatment on right

Examples of endoscopic images of Barrett’s oesophagus before treatment with the 360 RFA Express Catheter and at first follow up endoscopy are shown in Fig. 3.

47 patients had reached End of Treatment (EoT) biopsies as per protocol seen in Fig. 1. 40/47 patients (85%) had complete resolution of dysplasia (CR-D) and 34/47 (76%) had complete resolution of intestinal metaplasia (CR-IM). The median number of focal RFA treatments following the index RFA Express treatment was 2 (IQR 1–4) to reach EoT. The treatment protocols used are shown in Table 3.

**Table 3:** Treatment protocols used

Treatment Protocol	No of patients (%)
10 J/cm^2^/no clean/10 J/cm^2^	87 (78%)
10 J/cm^2^/clean/10 J/cm^2^	18 (16%)
12 J/cm^2^/no clean/12 J/cm^2^	7 (6%)

17/112 patients (15%) developed oesophageal strictures that were symptomatic, and which required endoscopic dilation. The median number of dilations needed to resolve these was 2 (IQR 2–4). 2 patients required 5 dilations. None formed refractory strictures.

**Fig. 4:**
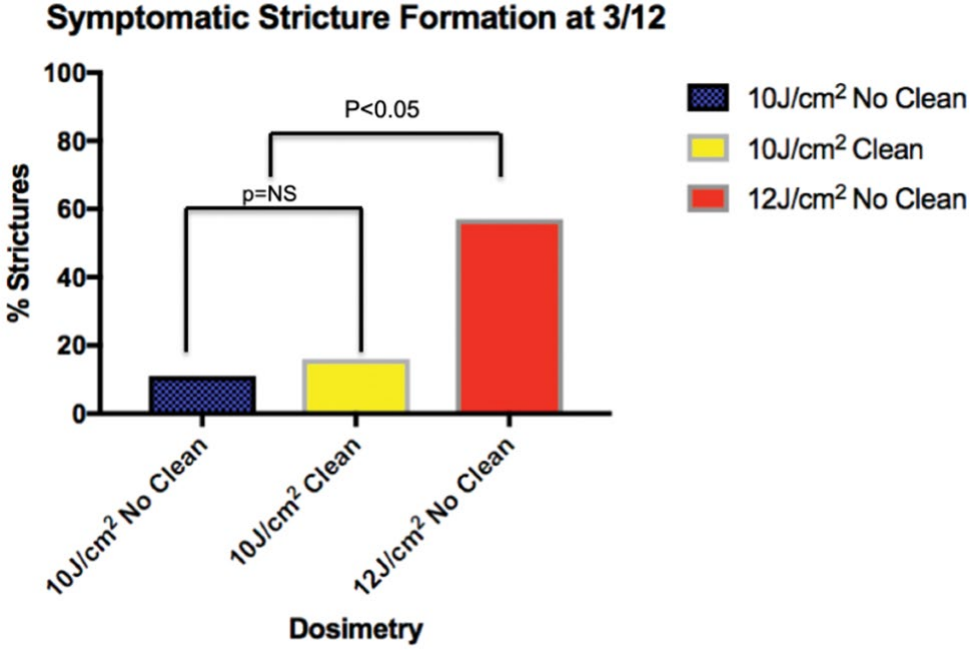
Percentage of patients with each regimen who developed a symptomatic stricture at 3 months post RFA Express treatment

Secondary analysis was performed to assess if stricture formation was related to the treatment regimen used. 10/87 (11%) patients treated with 10 J/cm^2^/no clean/10 J/cm^2^ developed strictures. 3/18 (16%) patients treated with 10 J/cm^2^/clean/10 J/cm^2^ developed a stricture. 4/7 (57%) patients treated with 12 J/cm^2^/no clean/12 J/cm^2^ developed a stricture. There was no significant difference seen between 10 J/cm^2^/clean/10 J/cm^2^ and 10 J/cm^2^/no clean/10 J/cm^2^ p = NS. (Fig. 4) The 12 J/cm^2^ energy setting was associated with significantly more strictures than the 10 J/cm^2^ Fisher’s Exact test P < 0.05. There was no significant difference in the number of dilations required across the energy settings and techniques. The 2 patients who required 5 dilations were in the 10 J/no clean/10 J/cm^2^ group.

**Fig. 5:**
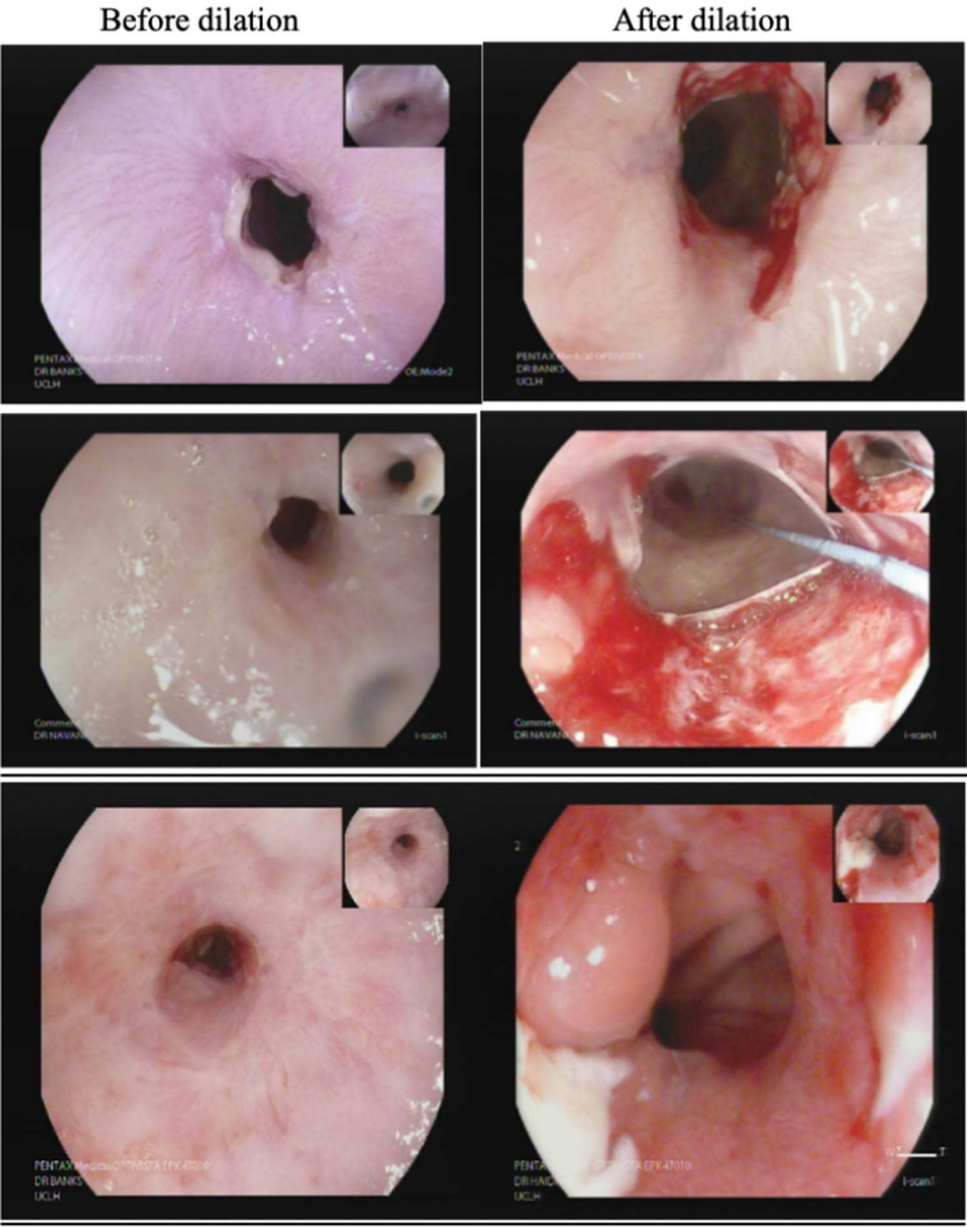
Endoscopic images of post RFA Express strictures before and after dilation

Endoscopic images of strictures formed after RFA Express treatment, and following endoscopic dilation are shown in Fig. 5.

## Discussion

This study represents real world data on the initial use of the HALO 360 Express catheter in the UK and Ireland with different endoscopists in different centres.

The percentage reduction in visible Barrett’s mucosa at first follow up endoscopy at 3 months demonstrates effective, rapid squamous re-epithelialisation of the Barrett’s mucosa after a single treatment with the 360 Express balloon. It is therefore a good quantitative measure of assessing the total BE surface area that has been successfully ablated following optimum catheter and electrode contact at index treatment. However, a limitation is that changes in C and M may not represent fully the circumferential area change following ablation as the maximal length may vary from a small percentage of the circumferential surface area to a larger area. This method was chosen due to the retrospective nature of the study with images not available from all follow up endoscopies to allow an overall assessment of regression.

In our study we show that 78% ± 16 of the circumferential length of the mucosa was re-epithelialised following a single RFA Express treatment and 55% ± 36 of the maximal length was re-epithelialised following a single RFA Express treatment. However, the clinical significance of this is uncertain. Furthermore in those patients with a more complex oesophagus with variable OID, the contact may not be so good with a circumferential device. Although an ANOVA was performed to assess regression across treatment regimens, this was non-significant although this is likely due to the small numbers in two of the regimen groups.

We demonstrate that in the 47 patients who had reached end of treatment (EoT) biopsies 85% had achieved complete resolution of dysplasia (CR-D) and 76% had achieved complete resolution of intestinal metaplasia (CR-IM). This is comparable to other series [17–19] (Table 4) although the number of patients who have reached EoT biopsies is fewer than half of the patients in the study.

**Table 4:** Previous results from other series of stepwise eradication of Barrett’s oesophagus with ER and RFA

	Complete resolution of dysplasia (CR-D)	Complete resolution of intestinal metaplasia (CR-IM)	Stricture rate	Average number of dilations to overcome stricture	Average number of treatments needed per patient
Shaheen et al. [7]	90.5%	77.4%	6%	2.6	3.5
Haidry et al. [17]	81%	62%	9%	1.3	2.5
Haidry et al. [18]	92%	83%	6.2%	2	2.5
Phoa et al. [19]	92.6%	88.2%	11.8%	1	3
Phoa et al. [20]	92%	87%	6%	1	3

The overall stricture rate in the patients in our cohort is 15% and appears to be somewhat higher than that of other series with the old HALO 360 catheter [10, 17–20] which have reported rates of 6–10%. The median number of dilations required was 2 and although 2 patients required 5 dilations, most were not refractory strictures and were therefore amenable to endoscopic balloon dilatation to allow alleviation of the resultant dysphagia. There was a statistically significant increase in the rate of stricture formation with 12 J energy rather than 10 J but not between the clean and no clean regimens, but due to the retrospective nature of the analysis it can be difficult to interpret this difference between groups as the study was not powered to do this. It is not possible to comment if more refractory strictures were formed with the different energy levels and techniques due to the numbers being small. Although the 2 patients who required 5 dilations were in the 10 J/no clean/10 J/cm^2^ group this may be due to the higher overall number of strictures in this group. We consider the most important finding to be the comparison with other series looking at the previous HALO 360 system, although clearly this is not a head-to-head randomised comparison.

The patient numbers are low, and the study was not controlled or powered to show a difference in the stricture rates between the dosimetry and cleaning regimens. It would be logical however that no routine use of a protocol in this study might have an impact on the outcomes in terms of both resolution of dysplasia and stricture formation. This is a weakness of our data which resulted from the retrospective nature of the study. Indeed on discussion of the data between authors, some noted how different their protocols were to others. However, on the basis of these, and a recently completed randomised trial from another centre [21], the HALO registry of UK and Ireland has already distributed advice to centres to use the manufacturer’s approved settings of 10 J/cm^2^/clean/10 J/cm^2^. Interestingly this study [21] demonstrated an unreasonably high stricture rate of 21% in the 10 J/cm^2^/no clean/10 J/cm^2^ regimen which was much higher than that seen in our study. Data are currently being collated with this standardised treatment protocol to report efficacy data and stricture rates in a subsequent cohort of patients by our group. It is hoped that the unified protocol of energy levels, washing and cleaning will lead to clearer outcome data and fewer strictures.

The reason for the observed higher rate of stricture formation with this balloon in our study is unclear. It may be that the improved tissue contact with the self-sizing balloon allows easier and deeper transmission of energy from the balloon electrodes when compared to the original device which was sized for the narrowest diameter of the oesophagus. Furthermore the larger balloon may increase the risk of overlap of ablations. In addition, the cleaning step which was originally introduced to improve transmission of energy may allow “cooling” of the mucosa between energy applications preventing deeper transmission of energy from the balloon catheter. By eliminating these and performing sequential ablations there may be a deeper depth of injury or no time for the heat caused by the initial ablation to dissipate prior the second ablation causing an additive build up of energy transmission and thermal injury with deeper tissue permeation causing submucosal injury and stricture formation. However, this is not shown by our data. It may also be that post endoscopic resection strictures are more likely as the balloon size is not reduced in these patients as with the previous device.

The strengths of this study are that it demonstrates the use of the 360 Express catheter in a real-world setting across multiple specialist centres and with multiple different endoscopists, which we feel enhances the clinical relevance of our findings as may mean the outcomes are more applicable to different centres as opposed to single centre studies with a very small number of endoscopists. In addition, there was a broad range of index patient characteristics of the baseline Barrett’s mucosa with a range of highest baseline pathology and patients who had either received endoscopic resection before entering an RFA treatment protocol, and those who had not. This may allow for some generalisability of the results.

The limitations of the study are that fewer than half the patients have reached end of study biopsies. Prospective data are being collected on subsequent patients by our group. In addition, different treatment protocols were used so it is difficult to know if the stricture rate of the 360 Express balloon is higher than that of the original 360 balloon if the manufacturer’s recommended treatment protocol of 10 J/cm^2^/clean/10 J/cm^2^ is used. In addition, although the EoT rates are similar to other studies [10, 17–20], more patients will be required in future to assess if the number of treatments is less to reach CR-D and CR-IM.

The retrospective nature of this study meant we were not able to accurately analyse number of intubations and duration of the procedures which are some of the suggested benefits of the Express catheter although reduced duration has been reported elsewhere [15]. As mentioned above we used C and M to assess BE regression rather than, for example, examination of endoscopic images to allow inclusion of those patients for whom, for example, images were not available. We did not record other complications including pain or bleeding as this information would not be recorded for all patients and we wanted to demonstrate consecutive real world data of all patients treated by early adopters of the HALO Express system. In addition, this study was not powered to show a difference in the secondary outcome of stricture rates, being an observational, descriptive study.

In conclusion, further work is needed prospectively to assess the number of treatments needed to reach CR-D and CR-IM, the stricture rate with 10 J/cm^2^/clean/10 J/cm^2^ and the duration of procedures and post procedural metrics such as patient pain scores and satisfaction with the proposed reduced number of intubations. However, the Express device in a real world setting appears to have favourable surface area regression.
